# Sensitive Immunochromatographic Determination of *Salmonella typhimurium* in Food Products Using Au@Pt Nanozyme

**DOI:** 10.3390/nano13233074

**Published:** 2023-12-04

**Authors:** Olga D. Hendrickson, Nadezhda A. Byzova, Irina V. Safenkova, Vasily G. Panferov, Boris B. Dzantiev, Anatoly V. Zherdev

**Affiliations:** A.N. Bach Institute of Biochemistry, Research Center of Biotechnology of the Russian Academy of Sciences, Leninsky Prospect 33, 119071 Moscow, Russia; odhendrick@gmail.com (O.D.H.); nbyzova@inbi.ras.ru (N.A.B.); saf-iri@yandex.ru (I.V.S.); panferov-vg@mail.ru (V.G.P.); dzantiev@inbi.ras.ru (B.B.D.)

**Keywords:** *Salmonella typhimurium*, immunochromatographic analysis, nanozyme, foodborne pathogens, food safety

## Abstract

In this study, we developed a sensitive immunochromatographic analysis (ICA) of the *Salmonella typhimurium* bacterial pathogen contaminating food products and causing foodborne illness. The ICA of *S. typhimurium* was performed using Au@Pt nanozyme as a label ensuring both colorimetric detection and catalytic amplification of the analytical signal due to nanozyme peroxidase-mimic properties. The enhanced ICA enabled the detection of *S. typhimurium* cells with the visual limit of detection (LOD) of 2 × 10^2^ CFU/mL, which outperformed the LOD in the ICA with traditional gold nanoparticles by two orders of magnitude. The assay duration was 15 min. The specificity of the developed assay was tested using cells from various *Salmonella* species as well as other foodborne pathogens; it was shown that the test system detected only *S. typhimurium*. The applicability of ICA for the determination of *Salmonella* in food was confirmed in several samples of milk with different fat content, as well as chicken meat. For these real samples, simple pretreatment procedures were proposed. Recoveries of *Salmonella* in foodstuffs were from 74.8 to 94.5%. Due to rapidity and sensitivity, the proposed test system is a promising tool for the point-of-care control of the *Salmonella* contamination of different food products on the whole farm-to-table chain.

## 1. Introduction

A growing global population and increased food needs lead to the intensification and industrialization of the crop and livestock sectors, which creates not only new opportunities but also new threats and risks to food safety [1]. These threats include foodborne illnesses—infectious diseases or intoxications caused by bacteria, viruses, or parasites that enter the body through contaminated water or food [2]. Foodborne diseases are not only harmful to human health but are a major impediment to socioeconomic development as they put pressure on health systems and harm the national economies, tourism, and trade sectors [3]. One of the relevant foodborne pathogens is *Salmonella*—a Gram-negative rod-shaped enterobacterium [4]. The main symptoms of salmonellosis include fever, headache, nausea, vomiting, abdominal pain, and diarrhea [4]. Foodstuffs associated with *Salmonella* outbreaks include milk, domestic animal meat, poultry, eggs, and other animal products [5,6]. *Salmonella* can pass through the entire food chain—from animal feed and primary production to the home or food service establishments—and persist in food and the environment for a long time. One of the highly pathogenic *Salmonella* species is *Salmonella typhimurium*, which causes infectious diseases with intestinal symptoms [7,8].

The serious consequences of diseases caused by *Salmonella* make it necessary to prevent, identify, and eliminate the risks associated with contaminated food. Prevention of foodborne infections requires control measures at all stages of the food chain—from agricultural production to food processing, production, and cooking. Early detection and adequate response to salmonellosis will prevent its further spread and ensure food safety. The traditional methods for detecting *Salmonella* cells include microbiological (cultivation of the test material on nutrient media) and microscopic (study of stained material) techniques [9,10]. Although these approaches provide effective diagnostics, they are time- and labor-intensive and not applicable in the context of quick monitoring of many samples for the presence of pathogens. Another modern method for detecting bacterial pathogens is molecular genetic analysis [9]. Despite the high sensitivity and accuracy, this approach requires high-tech equipment, qualified specialists, and special reagents.

Alternative analytical methods are those based on the immunoanalytical detection of foodborne pathogens using specific antibodies, first of all, enzyme-linked immunosorbent assay (ELISA), which provides sensitive and selective detection of bacteria, including *Salmonella* [11,12,13,14]. However, a typical ELISA contains several steps and requires some laboratory equipment; therefore, it cannot be classified as a rapid on-site method. Immunochromatographic analysis (ICA), which is widely used for the detection of pathogens of various foodborne infections does not have this disadvantage [15,16]. The merits of ICA are the simplicity of the analytical procedure, rapidity, and low cost. All reagents are preliminarily applied to the test strip and the analysis consists of a simple incubation of the strip with the tested sample followed by a visual qualitative (yes/no) or instrumental quantitative (how much) assessment of the pathogen. In addition, ICA often does not require complex sample preparation before analysis, and for liquid food samples, simple dilution with buffer is often carried out, which contributes to the rapidity of testing [17,18].

Thus, for rapid and sensitive mass control of *Salmonella* in food, ICA is a promising and demanded method, which is evidenced by a large number of relevant publications (see Section 3.6). On average, the detection sensitivity of *Salmonella* is 10^4^–10^6^ CFU/mL [19,20,21]. In several studies, the authors use special approaches to reduce the limit of detection (LOD) by using new markers that can enhance the detected signal [22] including using single-atom nanozymes [23], fluorescent labels [24,25], aggregation or extension of labels [26,27], etc. In this case, LODs decrease to 10^2^–10^3^ CFU/mL. Among the tested matrices, mainly liquid samples (milk, drinking water, juice) can be mentioned, the assay duration varies from 5 min to several hours.

In the present study, an enhanced ICA of *S. typhimurium* was for the first time developed in a sandwich format using core@shell bimetallic Au@Pt nanozyme as a label gaining high popularity due to high activity and enzymatic specificity along with high stability, and low cost [28,29]. The catalytic properties of the marker allowed for the amplification of the optical signal and a significant reduction in the LOD of *S. tiphymurium* was achieved. The assay was validated for *S. typhimurium* detection in a panel of food samples—milk and chicken meat.

## 2. Materials and Methods

### 2.1. Reagents and Materials

Gold (III) chloride hydrate (HAuCl_4_ × H_2_O), sodium hexachloroplatinate (IV) hexahydrate (Na_2_PtCl_6_ × 6H_2_O), N-hydroxysuccinimide ester of biotin, sodium citrate, sodium azide, sodium ascorbate, bovine serum albumin (BSA), sucrose, Tris, Tween-20, Triton X-100, 3,3′,5,5′-tetramethylbenzidine dihydrochloride (TMB), 30% hydrogen peroxide, dimethyl sulfoxide (DMSO), and streptavidin conjugated with horseradish peroxidase (STR–HRP) were from Sigma-Aldrich (St. Louis, MO, USA). A chromogenic substrate kit for peroxidase activity based on 3,3′-diaminobenzidine (DAB) was purchased from Servicebio (Wuhan, China). The monoclonal antibodies (MAb) to lipopolysaccharide (LPS) of *S. typhimurium*, clone 1E6cc, were from HyTest (Moscow, Russia). Goat anti-mouse immunoglobulins (GAMI) were from Arista Biologicals (Allentown, PA, USA). All other chemicals were purchased from Khimmed (Moscow, Russia); they were of analytical grade and were used without further purification. All solutions were prepared with ultrapure water with a resistivity of 18.2 MW (Millipore Corporation, Burlington, MA, USA).

The following bacterial strains (with concentrations of their standard solutions indicated in brackets) were obtained from the State Collection of Pathogenic Microorganisms and Cell Cultures “GKPM–OBOLENSK” (Obolensk, Moscow region, Russia): *S. typhimurium* (2.3 × 10^9^ CFU/mL), *S. enteritidis* 3–2 (3.1 × 10^9^ CFU/mL), *S. paratyphi* A56 (2.8 × 10^9^ CFU/mL), *S. virchov* 06 (2.4 × 10^9^ CFU/mL), *S. anatum* 1120 (1.5 × 10^9^ CFU/mL), *Escherichia coli* 0157:H7 ATCC51658 (2 × 10^9^ CFU/mL), *Listeria monocytogenes* ATCC51658 (5 × 10^9^ CFU/mL), *Yersinia enterocolitica* H-26-04 (1 × 10^9^ CFU/mL), *Yersinia pseudotuberculosis* 4320 (1 × 10^9^ CFU/mL), *Pseudomonas aeruginosa* ATCC27853 (1 × 10^9^ CFU/mL), and *Francisella tularensis holarctica* 15 (5 × 10^9^ CFU/mL). Concentrations of pathogens were measured by the collection holder using microbiological tests.

A nitrocellulose working membrane (CNPC-SS12, having a 15 µm pore size), a glass fiber conjugate pad (PT-R7), a sample pad (GFB-R4), and an adsorbent pad (AP045) were purchased from Advanced Microdevices (Ambala Cantt, India). The 96-well transparent polystyrene microplates for the ELISA were purchased from Corning Costar (Tewksbury, MA, USA).

### 2.2. Biotinylation of MAb

MAb were biotinylated according to [30]. For this, the MAb solution (100 μM, 200 μL) in 50 mM K-phosphate buffer with 100 mM NaCl, pH 7.4 (PBS) was mixed with a solution of N-hydroxysuccinimide ester of biotin (1 mM in DMSO) and incubated for 2 h at room temperature with continuous stirring. Excess unreacted low molecular weight reagents were removed by the dialysis against PBS.

### 2.3. ELISA of S. typhimurium

MAb (1 μg/mL, 100 μL in PBS) were immobilized in the microplate wells overnight at 4 °C. Then, the microplate was washed four times with PBS containing 0.05% Triton X-100 (PBST). Next, solutions of *S. typhimurium* (concentration range from 1 × 10^7^ to 1 × 10^4^ CFU/mL, 50 μL in PBST) and biotinylated MAb (1 μg/mL, 50 μL in PBST) were added to the wells. The microplate was incubated for 1 h at 37 °C and washed as described above. After that, STR–HRP (1:5000 dilution, 100 μL in PBST) was added to the wells and incubated for 1 h at 37 °C. After washing, the activity of the enzyme label was determined. To do this, 0.4 mM TMB solution in 40 mM sodium citrate buffer, pH 4.0, containing 3 mM hydrogen peroxide (100 μL) was added to the microplate wells and incubated for 15 min at room temperature. The reaction was stopped by adding 1 M sulfuric acid (50 μL) and the optical density was measured at 450 nm (OD_450_) on a Zenyth 3100 microplate spectrophotometer (Anthos Labtec Instruments, Wals, Austria).

### 2.4. Synthesis and Characterization of AuNPs and Au@Pt Nanozyme

Synthesis of two preparations of AuNPs (of smaller and larger diameters) was performed following the procedure of HAuCl_4_ reduction by sodium citrate [31]. To obtain smaller/larger AuNPs, HAuCl_4_ (97.0/98.8 mL of 0.01% solution) was heated to 100 °C and then, sodium citrate (3.0/1.2 mL of 1% solution) was added. The solutions (OD_520_ = 1) were kept at 100 °C for 20/30 min under vigorous stirring and then cooled.

Au@Pt was synthesized using AuNPs with a smaller diameter as described by Gao et al. with modifications [32]. AuNPs (40 mL) were mixed with 10 mM Na_2_PtCl_6_ (8 mL) and deionized water (10.6 mL). The resultant solution was heated in a water bath to 80 °C. After that, 50 mM sodium ascorbate (8 mL) was added with a flow rate of 450 μL/min using a peristaltic pump. After heating the reaction mixture for another 20 min at 80 °C and cooling it to room temperature, the resultant Au@Pt was centrifuged at 12,000× *g* for 20 min. The sediment was resuspended in deionized water.

To characterize the size and aggregation of the obtained nanodispersed labels, transmission electron microscopy (TEM) was utilized. AuNPs and Au@Pt nanozyme were applied to 300-mesh grids (Pelco International; Redding, CA, USA) coated with a poly(vinyl formal) film. The images were obtained with a JEM CX-100 microscope (Jeol, Tokyo, Japan) at 80 kV and analyzed by the Image Tool 3.00 software (University of Texas, Health Science Center, San Antonio, TX, USA).

For elemental analysis, energy-dispersive spectroscopy (EDS) of Au@NPs and Au@Pt was performed. For this, the transmission electron microscope Jeol JEM-1400 (Jeol, Tokyo, Japan) equipped with the energy-dispersive spectrometer (INCA Energy TEM 350, Oxford Instruments, High Wycombe, UK) was utilized. AuNPs and Au@Pt were centrifuged twice (for 20 min at 20,000× *g* and 15,000× *g*, respectively) and redispersed in ultrapure water. The aliquots of AuNPs and Au@Pt (10 μL both) were incubated on a formvar-coated copper grid for 10 min. EDS spectra were recorded in an area of 0.5 μm^2^.

The complexation of Au@Pt with MAb was confirmed by the asymmetrical flow field-flow fractionation (AF4) coupled with UV-vis detector, multi-angle laser light scattering (MALLS), and dynamic light scattering (DLS). For this, AF4 platform that included Wyatt Eclipse 3+ Separation System with Eclipse Long Channel (Wyatt Technology, Goleta, CA, USA), 1260 Infinity LC System (Isocratic Pump, Autosampler, Variable Wavelength Detector, Agilent Technologies, Santa Clara, CA, USA), Dawn HELEOS II multi-angle light scattering detector with a WyattQELS DLS module, and Optilab T-Rex refractometer (Wyatt Technology, Goleta, CA, USA) was applied. The characterization of Au@Pt and MAb–Au@Pt conjugate was carried out according to [33]; the details on the liquid flow program are presented in Table S1. The data were collected and analyzed with ChemStation v.B.04.03 (Agilent Technologies, Santa Clara, CA, USA) and Astra v.6.1.7.17 (Wyatt Technology, Goleta, CA, USA) software.

### 2.5. Conjugation of MAb with AuNPs and Au@Pt

Before conjugation, MAb were dialyzed against 10 mM Tris-HCl buffer, pH 9.0, for 2 h at 4 °C, and AuNPs and Au@Pt nanozyme were adjusted to pH 9.0 by 0.1 M K_2_CO_3_. AuNPs/Au@Pt nanozyme were added to MAb solutions (4.2/20 μg/mL) and incubated for 30/60 min at room temperature with stirring. Then, an aqueous solution of BSA was added to the reaction mixtures to a final concentration of 0.25%. After that, Mab–AuNPs/MAb–Au@Pt were centrifuged at 17,000×/12,000× *g* for 15/20 min at 4 °C. The precipitates were concentrated (by 6 and 10 times for Mab–AuNPs and MAb–Au@Pt, respectively) by resuspending in small volumes of 0.01 M Tris-HCl, pH 9.0, containing 1% BSA, 1% sucrose, and 0.01% sodium azide (TBSA), and stored at 4 °C.

### 2.6. Production of Immunochromatographic Tests

Two types of test strips were produced to perform AuNPs-based and Au@Pt nanozyme-based ICAs. In the first case, the control (C) zone and the test (T) zone were formed on the working membrane by application of GAMI (0.5 mg/mL in PBS) and MAb (1 mg/mL in PBS), respectively, using an Iso-Flow dispenser (Image Technology, Lebanon, NH, USA) at a loading of 0.12 μL/mm of the membrane. MAb–AuNPs conjugate containing 1% of Tween 20 (OD_520_ = 6) was manually applied to the fiberglass conjugate pad (0.8 μL/mm).

In the second case, C and T zones were also formed by the immobilization of GAMI (0.5 mg/mL in PBS) and MAb (2 mg/mL in PBS). MAb–Au@Pt was 13 times diluted by TBSA containing 0.05% of Tween-20 and applied to the conjugate pad (with a loading of 32 μL/cm of the membrane). The same glass fiber membrane was used both as a sample and a conjugate pad. The conjugate pad was preliminary soaked by the PBS containing 1% of Triton X-100. All membranes and pads were dried at room temperature for 20 h. Then, multimembrane sheets were composed consisting of the working membrane, sample, conjugate, and absorbent pads. Finally, test strips (of 3.5 mm width) were obtained by cutting sheets with an automatic Index Cutter-1 guillotine (A-Point Technologies, Gibbstown, NJ, USA). Test strips could be stored at room temperature in zipper bags with desiccant (silica gel) for at least 3 months without changing the analytical performance of the ICA.

### 2.7. Sample Preparation before the ICA and ELISA

As real samples, cow lactose-free milk of 1.5% fat (sample 1), cow milk of 3.2% fat (sample 2), cow-baked milk of 4% fat (sample 3), and raw chicken meat (sample 4) were purchased in local supermarkets. Milk was diluted by PBS containing 0.1% of Triton X-100 (PBST_0.1_) 10, 20, and 25 times for samples 1–3, respectively. For chicken meat, a procedure of sample preparation based on that described in [34] was used. Briefly, the meat sample was minced and 5 mL of the extraction buffer (PBST_0.1_ containing 0.5 M KCl) was added to 250 mg of homogenized meat. The mixture was intensively shaken for 20 min and then left for 15 min to settle the solid component. The upper layer was used for the analysis.

### 2.8. ICA of S. typhimurium Using AuNPs

Test strips were placed horizontally and the *S. typhimurium* standard solutions (concentration range of 3 × 10^7^–1 × 10^3^ CFU/mL, 60 μL) were applied to the sample pads. After 10 min incubation at room temperature, test strips were scanned using a CanoScan 9000F scanner (Canon, Tochigi, Japan). The obtained digital images were processed by TotalLab TL120 software (Nonlinear Dynamics, Newcastle, UK) to measure the intensity of zones’ coloration.

### 2.9. Common and Enhanced ICAs of S. typhimurium Using Au@Pt Nanozyme

Test strips were immersed vertically in the standard solutions of *S. typhimurium* in PBST_0.1_ (concentration range of 2.3 × 10^8^–23 CFU/mL, 75 or 40 µL for the common and enhanced ICAs, respectively) or real samples (milk and chicken extracts) and incubated for 10 min at room temperature. Then, in the case of the common ICA, test strips were taken out and blotted. For the enhanced ICA, test strips were washed (by placing the bottom edge of the strip to 25 μL of PBST_0.1_ for 3 min) and the DAB-based substrate solution (1 µL) was added directly to the T zone and incubated for 2 min. After both ICA modes, the images of the test strips were obtained and processed as described above.

### 2.10. Evaluation of the ELISA and ICA Results and Statistical Analysis

To fit the dependencies of OD_450_ (for the ELISA) or color intensity of the T zone (for the ICA) (y) versus *S. typhimurium* concentrations (x), OriginPro 9.0 software from OriginLab (Northampton, MA, USA) was applied. The analytical performance was determined following [35]. In the ELISA, the instrumental LOD of *S. typhimurium* corresponded to 10% binding with the immobilized MAb. In the ICA, visual LOD was estimated as the minimum *S. typhimurium* concentration causing a visible coloration in the T zone. That is, the visual LOD was assessed as the concentration of the analyte at which the coloration of the T zone on the test strip is noticeable to the naked eye (usually this corresponds to approximately 600–700 RU and more). The linear range of the detectable *S. typhimurium* concentrations corresponded to the diapason where linear approximation allows obtaining reliable quantitative results.

All measurements were made in triplicate. For the ELISA and ICA calibration curves, the Means ± SE (standard errors) of *S. typhimurium* concentration were calculated. To evaluate the repeatability and reproducibility of the assay, intra-assay coefficients of variation (CV) and inter-assay CV were assessed (*n* = 6). For the analysis of milk and meat samples, 5 repeats were made for each sample.

## 3. Results and Discussion

### 3.1. Obtaining and Characterization of the Reagents

**Figure 1:**
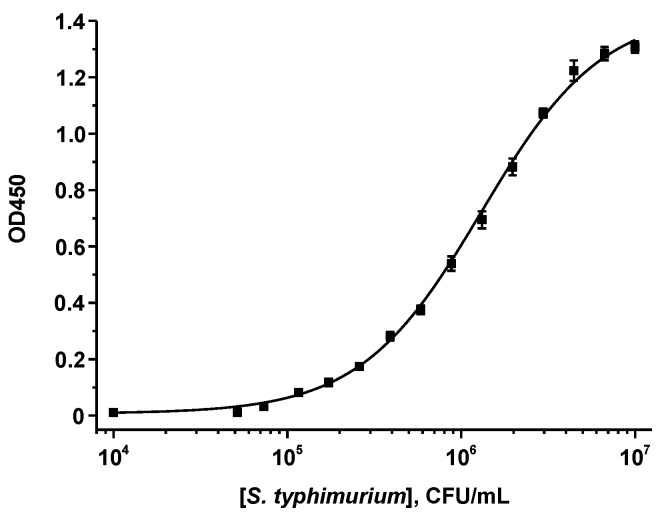
Calibration curve of *S. typhimurium* in the sandwich ELISA (*n* = 3).

The antigen-binding properties of *Salmonella*-specific MAb were characterized in the sandwich format of the ELISA, where the detected antigen occurs in a triple complex with antibodies of the same or different clones. Anti-*Salmonella* antibodies of the 1E6cc clone were used for immobilization on the solid phase and the same biotinylated antibodies in combination with a STR–HRP conjugate were used to label the resulting *Salmonella*–MAb complexes. For bacteria, the use of the same clone is acceptable due to the recurrence of antigenic determinants on the cell surface. Optimization of the ELISA included choice of the concentration of the immobilized and biotinylated Mab (the ELISA optimization results are presented in Figure S1; the varied and finally selected parameters—in Table S2). Finally, the following values were selected: Mab were absorbed at 1 µg/mL and biotinylated Mab was added at the concentration of 2 µg/mL. The calibration curve of *S. typhimurium* is shown in Figure 1. The LOD was 2.2 × 10^5^ CFU/mL and the linear range was 4.3 × 10^5^–4.0 × 10^6^ CFU/mL.

**Figure 2:**
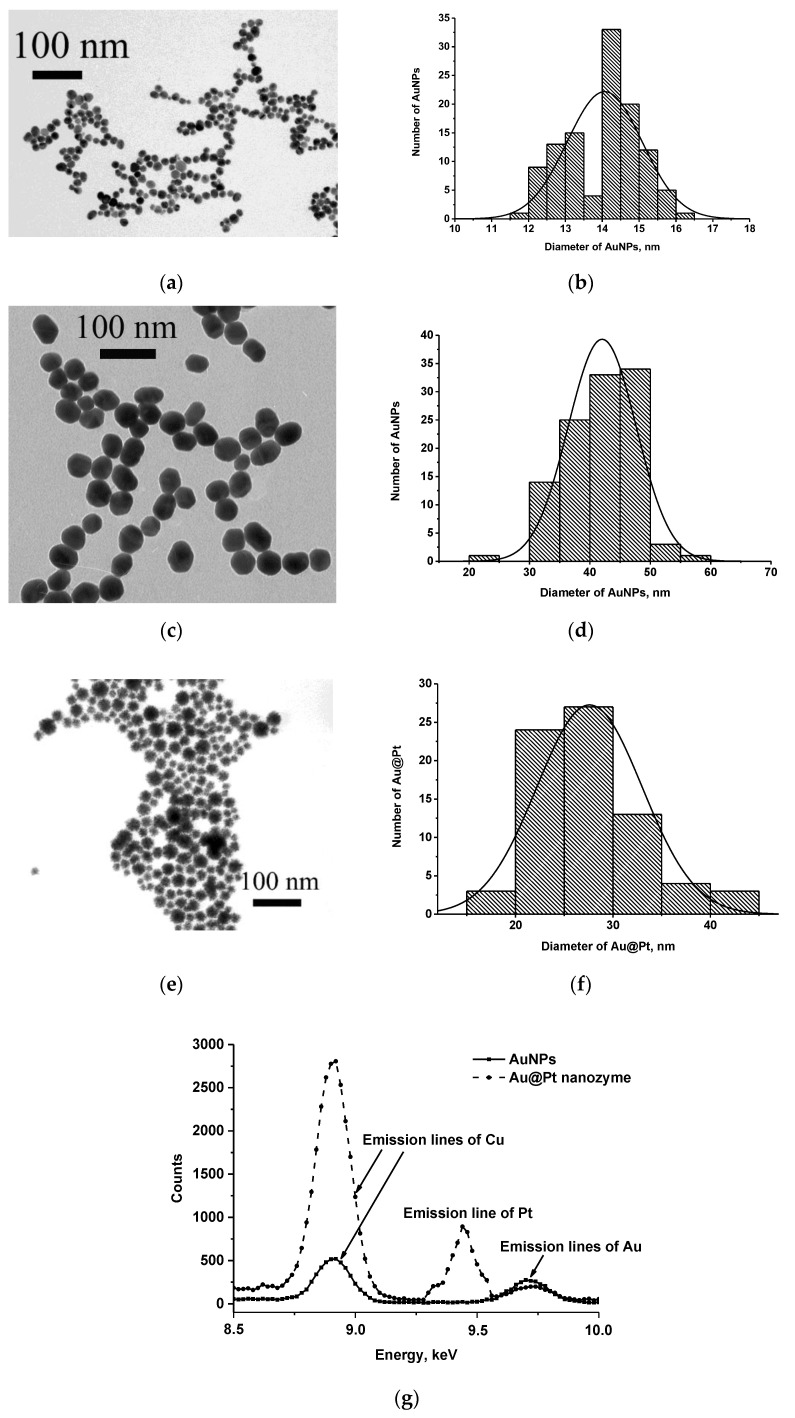
Microphotographs of AuNPs with smaller (**a**) and larger (**c**) diameters and Au@Pt nanozyme (**e**) and the corresponding histograms of size distribution (**b**,**d**,**f**) and EDS spectra of AuNPs and Au@Pt nanozyme (**g**).

Two types of nanodispersed carriers, AuNPs and Au@Pt nanozymes, were used as labels in the analyses. AuNPs are widely used in various ICA formats as an easily obtained and stable marker that provides a high and reproducible colorimetric signal on test strips [36]. Two preparations of AuNPs were synthesized by the citrate method varying the concentrations of the reagents and the incubation time for the preparation of larger and smaller particles. The size and homogeneity of AuNPs were characterized by TEM; the results are presented in Figure 2a–d. The observation showed that the shape of the AuNPs in both samples was close to spherical and no aggregates were found. Based on the electron microscopic images of AuNPs, the mean diameters/ellipticity coefficients were 14.1 ± 1.0 nm/1.1 ± 0.1 (according to measurements of 113 particles) and 42.0 ± 5.6 nm/1.3 ± 0.2 (according to measurements of 111 particles) for particles of smaller (Figure 2a,b) and larger (Figure 2c,d) diameters. A nanozyme label was obtained using AuNPs with a diameter of 14 nm by reducing the platinum salt with sodium ascorbate on a gold surface. Thus, core@shell Au@Pt nanoparticles with peroxidase-mimic catalytic properties were obtained. According to the TEM data (Figure 2e,f), the average size/ellipticity of Au@Pt particles was 27.1 ± 7.2 nm/1.2 ± 0.01 (according to measurements of 74 particles). Due to its black color, the nanozyme can form high-contrast lines on the test strip and can also be used as a colored label.

To confirm the composition of AuNPs and Au@Pt nanozyme, their elemental analysis was performed using EDS. The obtained spectra are presented in Figure 2f; they contain peaks characteristic of Au or/and Pt and, consequently, confirm the formation of objects composed of these elements. The detected peaks of copper refer to the copper grid used as a solid support for Au@Pt or AuNPs during EDS measurements.

**Figure 3:**
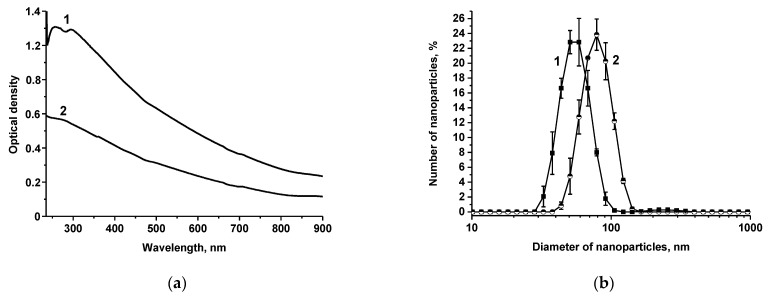
UV-Vis spectra of Au@Pt (1) and Mab–Au@Pt (2) (**a**) and the results of DLS measurements for Au@Pt (1) and Mab–Au@Pt (2) (**b**).

Conjugates of MAb with AuNPs and Au@Pt nanozyme were obtained by adsorption immobilization. The choice of MAb concentration for conjugation with 42 nm AuNPs was based on the dimensions of the carrier and immunoglobulin G (IgG) molecule and the maximum surface area occupied by one IgG molecule as described in [37]. Monolayer immobilization of MAb on the surface of AuNPs was ensured by the MAb concentration of 4.2 μg/mL. The choice of MAb concentration for conjugation with a nanozyme label was made based on our previous results; it was shown that for a nanozyme of such composition, the conjugate with an antibody load of 20 μg/mL was the most effective in the ICA [38]. Au@Pt and Mab–Au@Pt were characterized spectrophotometrically (Figure 3a) and by DLS on Zetasizer Nano ZS 90 (Malvern, UK) (Figure 3b). UV-vis spectra demonstrated a complete attenuation of the surface plasmon resonance band of gold after plating with platinum. According to the obtained DLS data, the diameters of Au@Pt and Mab–Au@Pt were ~55 nm and ~85 nm. The difference in the dimension values obtained by TEM and DLS is explained by the fact that DLS takes into account the hydration and protein shell on the surface of the studied particles. The zeta potentials of Au@Pt and Mab–Au@Pt were measured. The zeta potential of Au@Pt was −33.6 ± 0.5 mV, which correlated well with data obtained in other studies [39]. Conjugation of Au@Pt with Mab led to the converting of zeta potential to −8.17 ± 0.4 mV, which could be attributed to the positive charge of antibodies.

**Figure 4:**
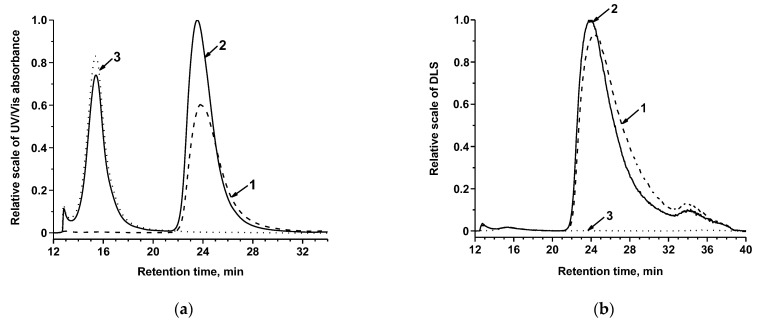
AF4 fractograms of Au@Pt–MAb conjugate at 400 nm (1) and 280 nm (2) and TBSA at 280 nm (3): UV-vis absorbance (**a**), DLS (**b**).

To confirm the conjugation between Au@Pt and MAb, the AF4 technique was used. UV-vis absorbance, MALLS, and DLS measurements were conducted at two characteristic wavelengths (280 nm—for the protein and nanozyme components and 400 nm—only for the nanozyme). The obtained AF4 fractograms are presented in Figure 4. As can be seen, the AF4 absorbance fractogram of Au@Pt–MAb conjugate and TBSA as its medium (which contained BSA) comprises the first peak (retention time is 15 min) of the conjugate at 280 nm due to TBSA (Figure 4a). The second peak (retention time is 23 min) of the conjugate corresponds to the absorbance at both 280 nm and 400 nm indicating the presence of Au@Pt. However, the absorption at 280 nm is much more intense than that at 400 nm (the peak area is smaller by ~25%) while the scattering for both fractograms was almost the same (Figure 4b). This confirms the presence of protein immobilized on the surface of the nanozyme. Au@Pt nanoparticles were not stable during fractionation and probably aggregated at the focusing stage.

### 3.2. ICA of S. typhimurium Using AuNPs

First, the ICA based on traditional AuNPs as a label was developed. In the sandwich format, the first capture antibodies are immobilized in the T zone. During the assay, the detected antigen from the test sample interacts with the second capture antibodies labeled by a marker and immobilized on the conjugate pad. The resulting complex moves to the T zone and binds there forming the first colored band. An excess of labeled antibodies moves on and binds to anti-species antibodies in the C zone to form a second colored band. Thus, in the presence of an analyte in the sample, a colored band occurs in the T zone and its intensity is directly proportional to the analyte concentration. In our case, both the first and second capture antibodies were of the same clone. To achieve the minimum LOD, the ICA was optimized by varying the concentrations of immunoreagents and the assay duration (Table S2). To form the T zone, the concentration of Mab was varied from 0.25 to 2 mg/mL. It was shown that the signal intensity increases in the concentration range of 0.25–1 mg/mL and remains constant at concentrations above 1 mg/mL, so the concentration of 1 mg/mL was chosen as the optimal one. For the formation of the C zone, the optimal concentration of GAMI was 0.5 mg/mL, which provided approximately the same intensity of the colorimetric signal in both zones at high cell concentrations. OD_520_ of the MAb–AuNPs conjugate was varied in the range of 1–8. In this interval, the signal intensity in the T zone increased, but at OD_520_ > 6, nonspecific background coloration was observed, therefore, OD_520_ = 6 was chosen as optimal. Labeled antibodies were applied with the loading of 16 µL/cm of the glass fiber membrane. This ensured the formation of intensely colored zones during the analysis combined with the complete washout of the reagent from the start of the strip and the absence of nonspecific coloration of the working membrane.

**Figure 5:**
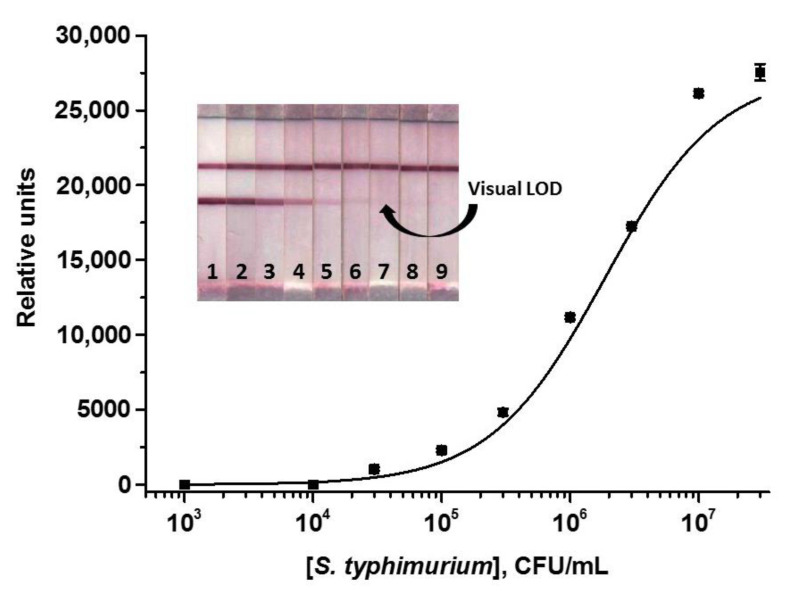
Calibration curve of *S. typhimurium* in the ICA with AuNPs and images of test strips after the assay. Concentrations of *S. typhimurium* are 3 × 10^7^ (1), 1 × 10^7^ (2), 3 × 10^6^ (3), 1 × 10^6^ (4), 3 × 10^5^ (5), 1 × 10^5^ (6), 3 × 10^4^ (7), 1 × 10^4^ (8), and 1 × 10^3^ (9) CFU/mL (*n* = 3).

The calibration curve of *S. typhimurium* cells obtained under optimized conditions is presented in Figure 5. The visual LOD for *S. typhimurium* cells was 3 × 10^4^ CFU/mL and the linear range was 4.5 × 10^5^–7.7 × 10^6^ CFU/mL.

### 3.3. Common and Enhanced ICAs of S. typhimurium Using Au@Pt Nanozyme

Common ICA with a nanozyme is similar to that with AuNPs. The only difference is the color of the bands formed on the test strip: in the case of a nanozyme label, bands have a shade from dark brown to black depending on the concentration of the conjugate. It should be noted that when using test strips in the usual configuration with a paper sample pad, nanozymes tend to stick to the sample pad. This effect was shown in our previous work [38] and confirmed in this study. Therefore, in order not to cut the composite under the lower edge of the working membrane, the type of the sample pad was changed, namely, instead of paper, a glass fiber membrane (the same as the conjugate pad) was used. Moreover, it was additionally impregnated with PBST_0.1_ to increase the mobility of the labeled conjugate and eliminate non-specific interactions. This replacement ensured the free movement of labeled antibodies along the test strip.

**Figure 6:**
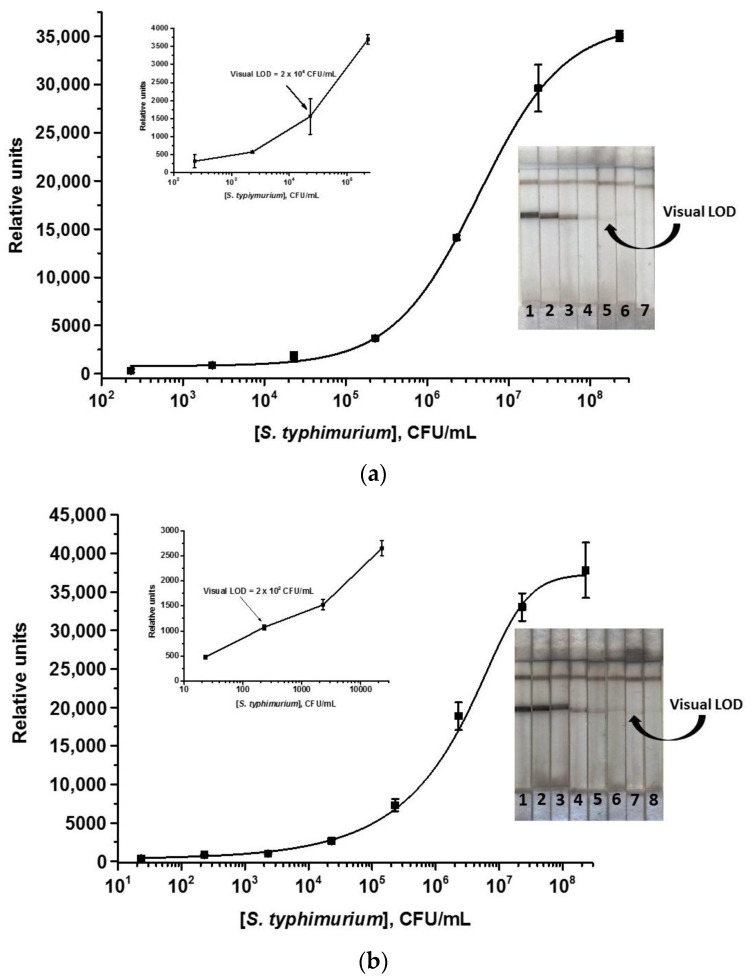
Calibration curves of *S. typhimurium* in the nanozyme-based common (**a**) and enhanced (**b**) ICAs, respectively, and images of test strips after the assay. Concentrations of *S. typhimurium* are 2.3 × 10^8^ (1), 2.3 × 10^7^ (2), 2.3 × 10^6^ (3), 2.3 × 10^5^ (4), 2.3 × 10^4^ (5), 2.3 × 10^3^ (6), 2.3 × 10^2^ (7), and 23 (8) CFU/mL (*n* = 3).

The optimization of the common ICA with nanozymes consisted of the selection of reagent concentrations and analysis time (see the varied and finally selected parameters of the ICA in Table S2). It was shown that an increase in the concentration of MAb immobilized in the T zone up to 2 mg/mL (concentrations from 0.75 to 2.5 were tested) led to the increase of the analytical signal and, consequently, the decrease of LOD of *S. typhimurium*. The GAMI concentration of 0.5 mg/mL was chosen from the interval of 0.2–0.6 mg/mL (less concentration led to a decrease in the intensity of the C zone while higher concentration resulted in no change in the color intensity). The dilution of the initial MAb–Au@Pt preparation for application to the conjugate pad was 1:13, which provided high-intensity bands and economical consumption of the reagent. A 10 min duration proved sufficient for the ICA; within this period, the entire volume of the reaction mixture (75 μL) was completely absorbed by the test strip, which also contributed to the saving of reagents while maintaining the maximum rapidity of analysis. The calibration curve of *S. typhimurium* obtained under optimized conditions is shown in Figure 6a. The *S. typhimurium* LOD was 2 × 10^4^ CFU/mL and the linear range was 8 × 10^5^–2 × 10^7^ CFU/mL. The estimated LOD was approximately the same as that of the AuNPs-based ICA.

The enhanced ICA was carried out under the same conditions as a common one with the addition of a catalytic enhancement step. Nanozyme having peroxidase-like properties can catalyze the oxidation reaction of the peroxidase substrate followed by the formation of an insoluble colored product. The additional coloration contributes to the enhancement of the colorimetric signal revealing those T zones that were invisible to the naked eye in the ICA without enhancement. Thus, an increase in the sensitivity of the determination of *S. typhimurium* is expected. In the enhanced ICA, the concentrations of reagents and the analysis time were the same as in the common ICA with nanozyme. The two most common peroxidase substrates, TMB and DAB, were used for the catalytic reaction. When applying TMB, non-specific coloration at the zero point (no *S. typhimurium* cells in the sample) and uneven background spotting of the entire test strip were observed. When using DAB, such undesirable side effects were absent but the volume and location of the substrate applied to the test strip were critical. In addition, a short washing step was added to eliminate residues of labeled antibodies on the working membrane and, accordingly, possible background coloration. It was shown that when the strip was washed for 3 min with buffer and a minimum volume of DAB (1 μL) was applied exactly to the T zone, the coloration of exactly the T zone was observed and no background signal at the zero point was registered. The standard curve of *S. typhimurium* in the enhanced ICA is shown in Figure 6b. As can be seen, the amplification stage enabled an increase in the signal intensity and reduction of the *S. typhimurium* LOD by two orders of magnitude down to 2 × 10^2^ CFU/mL. The linear range was 3.2 × 10^5^–1.2 × 10^7^ CFU/mL.

For the developed ICA, intra-assay and inter-assay CV did not exceed 15% in the linear range of *S. typhimurium* concentrations. The study of the stability of the test strips demonstrated that the functionality of the test system was retained after its storage for at least 3 months at room temperature.

### 3.4. ICA Specificity

The specificity of the test system was studied by analyzing other *Salmonella* strains (*S. paratyphi* A56, *S. virchov* 06, *S. enteritidis* 3–2, *S. anatum* 1120), as well as other relevant foodborne pathogens—*Escherichia coli* 0157:H7 ATCC51658, *Listeria monocytogenes* ATCC52658, *Yersinia enterocoliytica* H-26-04, *Yersinia pseudotuberculosis* 4320, *Pseudomonas aeruginosa* ATCC27853, and *Franciella tularensis holarctica* 15. According to the results obtained, no T zone coloration was observed in all cases although high concentrations of cells were used for the analysis (10^7^ CFU/mL) (Figure S2a); cross-reactivity values were <0.01%. Additional control experiments included the use of nanozyme conjugated with antibodies of another specificity (namely, to the myoglobin) and unmodified nanozyme instead of anti-*Salmonella* MAb–Au@Pt. In these cases, coloration in the T zones was also not observed (Figure S2b). Thus, the specificity of the test system only toward *S. typhimurium* cells was confirmed, and the absence of non-specific interactions was proved.

### 3.5. ICA of S. typhimurium in Cow Milk and Chicken Meat

As mentioned above, dairy products and meat from domestic animals and poultry are most often contaminated with *S. tiphymurium* among other foodstuffs. Accordingly, to validate the test system for the detection of *S. tiphymurium* cells, three samples of milk with different fat content (1.5, 3.2, and 4%) and chicken meat were purchased. One of the advantages of the ICA is the absence of complex sample preparation especially when testing liquid samples (milk, juices, drinking water, etc.) [40,41,42]. Before analysis, liquid samples are often elementarily diluted with a buffer to a dilution at which the detection will maximally correspond to that in the buffer.

Upon testing *Salmonella* cells in undiluted milk, some signs of the multi-component matrix influence on the assay performance were identified. First of all, a decrease in the coloration intensity in the T zone was observed because labeled antibodies stuck to membrane carriers or matrix components non-specifically blocked interacting reagents and prevented contacts for immune binding. Moreover, a background coloration of the working membrane was registered. Therefore, different regimes of milk dilution were tested as sample preparation. The criterion for the selection was the maximum match with the *S. typhimurium* calibration curve in the buffer (maximum optical signal value and LOD of the analyte). For milk samples with a high-fat content (3.2 and 4%), dilutions of 1:5, 1:10, 1:20, or 1:25 with PBST_0.1_ were tested. It was shown that the abovementioned criteria were met at milk dilutions of 1:20 for 3.2% milk and 1:25—for 4% milk (Table S3). For low-fat milk, 1:10 dilution (except it, we tested 1:5 dilution with PBST_0.1_) was sufficient (Table S3). That is, after the selected sample preparation, the fat component, which highly likely makes the greatest contribution to the total matrix effect, was equalized in all samples (to ~0.15%). Upon the selected conditions, the matrix value was leveled out.

For sample preparation of chicken meat, we used the approach earlier proposed for processing various types of meat before the detection of species-specific IgG [34]. This method is suitable in our case because sample preparation is not associated with heat treatment, as a result of which cells can lose their integrity and, consequently, immune recognition by specific antibodies can be impaired. Overall, meat sample preparation was short and simple, taking only 35 min. Therefore, the time of the enhanced ICA was 15 min; testing including sample preparation took 18 min for milk and 50 min—for chicken meat (from obtaining a sample to evaluating the results). Such assay duration fully meets the requirements for rapidity and allows for rapid mass screening of milk and meat samples.

To determine recoveries, 2 concentrations of *S. typhimurium* were used selected from the linear range, namely, 6 (or 7—for chicken meat) × 10^6^ and 2 × 10^7^ CFU/mL. The measured values (calculated in CFU per 1 g of food product) are presented in Table 1. As can be seen, the developed test system allows for the detection of 74.8–94.5% of *S. typhimurium* cells in milk and chicken meat.

For the ELISA, samples were additionally 10-fold diluted to fit the linear range.

The recovery values estimated by the ICA were compared with those obtained by the ELISA as a reference method. As can be seen from Table 1, good convergence of the ICA and ELISA results was observed, which confirms the accuracy of the developed method in detecting *Salmonella* cells in real samples.

### 3.6. Comparison with Other Studies

Table 2 presents the literature data on the ICA of *Salmonella* cells, sorted according to the need for additional separation/detection equipment. As can be seen, the first developments in the immunochromatographic determination of *Salmonella* appeared more than ten years ago. As expected, the assays were based on the use of such a traditional marker as AuNPs and were characterized by rather high LODs—from 10^4^ to 10^8^ CFU/mL [43,44]. AuNPs continued to be used in subsequent studies without significant progress in the sensitivity of *Salmonella* detection [19,21,45,46]. As the method evolved and different approaches were designed and applied to amplify the analytical signal, the analytical performance of ICAs of *S. typhimurium* was improved. Thus, the use of liposomes with encapsulated sulforhodamine [24], lanthanide label combined with immunomagnetic separation [47], the effect of increasing the concentration of the label due to salt-induced aggregation [27], increased signal based on gold growth [26], signal amplification in the SERS-based ICA [48], and ICA with MoS_2_ or graphene as labels [49] allowed reducing LODs down to 10^3^ CFU/mL (less often—down to 10^2^ CFU/mL). However, in some studies, such new approaches and manipulations as the use of multifunctional labels [20,50] or magnetic particles [51] did not completely give the desired effect leaving the assay sensitivity on par with that when using simple AuNPs (10^4^–10^5^ CFU/mL). Only a few complex approaches that require special detection equipment (i.e., SERS signal enhancement) or difficult-to-obtain composite labels have reduced the LOD down to 10^2^—tens of CFU/mL [28,52,53].

The objective of our research was to create an analytical system based on a new combination of nanozyme/detected analyte, which, in contrast to the works presented in the literature (see Table 2), would be characterized by (1) maximum simplicity of the analytical procedure, (2) non-instrumental detection, (3) simple pre-treatment of real samples, (4) maximum rapidity, (5) high specificity for *S. typhimurium*, and (6) possibility to detect the pathogen in a panel of detected samples (both liquid and solid). Consequently, obtaining the label with new properties was fast and unsophisticated. Au@Pt nanozyme was easily synthesized and ensured an intense reproducible signal. The analytical procedure was one step. No additional equipment was required for the detection of test results; cell presence could be estimated by the naked eye down to a concentration of 2 × 10^2^ CFU/mL. The specificity of the developed test system was very high: among 11 different pathogens of the *Salmonella* genus and other foodborne, only *S. typhimurium* could be revealed, which enabled the differentiation of this pathogen among numerous species that cause similar symptoms and clinical manifestations. Despite the introduction of additional steps of test strip washing and catalytic reaction, the analysis time was only 15 min. Moreover, ICA was implemented on an expanded panel of real samples (liquid and solid) including milk with different contents of fat and chicken meat. A simple sample preparation procedure provided sample processing during 3 min for milk (dilution with buffer) and 35 min for chicken meat processing.

The extended matrix panel essentially contributed to the characterization of this nanozyme as a marker because it was confirmed that the components of different real samples did not influence its functionality. As the additional functionality confirmation, the applicability of the MAb–Au@Pt conjugate for the detection of a bacterial polyvalent antigen was shown. In this case, a quite different manner of the assembly of labeled immune complexes than for low molecular weight or protein antigens (and, accordingly, a change in the flow process) can be noted. Therefore, detection of the foodborne pathogen seems not only a solution to a practical problem but also an additional assessment of the proposed nanomarker capabilities (which is often absent for new candidate developments, whose characterization is often limited to a single publication for a concrete analyte). The combination of precisely these characteristics favorably distinguishes the developed ICA from the reported works.

## 4. Conclusions

The enhanced ICA of *S. typhimurium* cells was developed using a nanozyme-based signal amplification. A 100-fold reduction in the LOD was achieved compared to common colorimetric ICAs based on AuNPs or a nanozyme. The food panel was tested using the developed assay; recoveries in the range of 74.8–94.5% were demonstrated. The proposed method seems to be promising and competitive due to the high sensitivity of *S. typhimurium* detection, rapidity, and proven applicability to various food products. It can be recommended for rapid mass control of many samples for the presence of the pathogen. The proposed approach, in which nanozymes catalytic properties are used for the development of the enhanced ICA, can be applied to the determination of any analytes.

## Figures and Tables

**Table 1:** Recoveries of *S. typhimurium* from cow milk and chicken meat (*n* = 5) estimated by the developed ICA and the ELISA as a reference method.

Added *S. typhimurium*, CFU/g	Revealed *S. typhimurium*, CFU/g	Recovery ± SD ^1^ (%)
ICA data		
Lactose-free milk with a fat content of 1.5%		
6 × 10^6^	4.5 × 10^6^ ± 0.4 × 10^6^	74.8 ± 7.2
2 × 10^7^	1.7 × 10^7^ ± 0.06 × 10^7^	82.5 ± 2.8
Milk with a fat content of 3.2%		
6 × 10^6^	5.1 × 10^6^ ± 0.2 × 10^6^	85.0 ± 2.9
2 × 10^7^	1.6 × 10^7^ ± 0.1 × 10^7^	78.8 ± 5.6
Baked milk with a fat content of 4%		
6 × 10^6^	5.0 × 10^6^ ± 0.3 × 10^6^	82.8 ± 5.6
2 × 10^7^	1.7 × 10^7^ ± 0.02 × 10^7^	86.0 ± 0.9
Chicken meat		
2.8 × 10^3^	2.6 × 10^3^ ± 0.07 × 10^3^	94.5 ± 2.4
0.8 × 10^6^	0.74 × 10^6^ ± 0.002 × 10^6^	92.2 ± 0.2
ELISA data		
Lactose-free milk with a fat content of 1.5%		
6 × 10^6^	5.5 × 10^5^ ± 0.4 × 10^5^	91.7 ± 7.7
2 × 10^7^	1.6 × 10^6^ ± 0.09 × 10^6^	80.0 ± 1.5
Milk with a fat content of 3.2%		
6 × 10^6^	5.2 × 10^6^ ± 0.25 × 10^6^	86.7 ± 4.2
2 × 10^7^	1.55 × 10^7^ ± 0.14 × 10^7^	77.5 ± 7.0
Baked milk with a fat content of 4%		
6 × 10^6^	5.4 × 10^6^ ± 0.3 × 10^6^	90.0 ± 5.0
2 × 10^7^	1.65 × 10^7^ ± 0.06 × 10^7^	82.5 ± 3.0
Chicken meat		
0.8 × 10^6^	0.65 × 10^6^ ± 0.04 × 10^6^	81.3 ± 5.0

^1^ Standard deviation, *n* = 3.

**Table 2:** Characteristics of the ICAs of *Salmonella*.

No	Detected Antigen	Label	LOD	Assay Duration, min	Matrix	Reference
Non-instrumental analysis						
1	*S. enteritidis*, *S. typhimurium*	AuNPs	10^4^, 10^6^ CFU/mL	5–15	Cell culture	[43]
2	*S. typhimurium*	Liposomes with encapsulated fluorescent dye	10^2^ CFU/mL	10	Buffer	[24]
3	*S. typhi*	AuNPs	1.14 × 10^5^ CFU/mL	15	Human serum	[44]
4	*S. typhimurium*	Liposomes with encapsulated fluorescent dye	1.2 cells/g	10–15	Tomatoes	[25]
5	*S. enteritidis*	AuNPs with signal amplification based on gold growth	10^4^ CFU/mL	360	Milk	[26]
6	*S. typhi*	AuNPs	Water: 3 × 10^8^ CFU/mL Milk: 3 × 10^7^ CFU/mL	10–15	Water and milk	[45]
7	*S. enteritidis*	MoS_2_ or graphene	10^3^ CFU/mL	10	Drinking water and watermelon juice	[49]
8	*S. enteritidis*, *S. *infantis**	AuNPs	10^6^ CFU/mL	4–5	Buffer	[19]
9	*S. typhimurium*	AuNPs	4 × 10^5^ CFU/mL	5–15	Chicken meat	[21]
10	*S. enteritidis*, *S. typhimurium*	Salt-induced aggregated AuNPs	10^3^ CFU/mL	14	Cabbage and drinking water	[27]
11	*S. gallinarum*, *S. pullorum*, *S. enteritidis*	AuNPs	Feces: 10^3^ CFU/mL; Meat: 10^2^ CFU/mL; Milk: 10^4^ CFU/mL	10	Fecal, meat, and milk	[46]
12	*S. typhimurium*	Au@Pt nanozyme with peroxidase-mimic activity	2 × 10^2^ CFU/mL	15	Milk of different fat content and chicken meat	This study
Analysis required additional equipment including that for magnetic separation						
13	*S. enteritidis*	Magnetic nanoparticles	1.95 × 10^5^ CFU/mL	30	Milk	[51]
14	*S. typhimurium*	Tris (dibenzoylmethane)mono(1,10-phenanthroline)europium(III) in polystyrene nanoparticles	10^3^ CFU/mL	90	Milk and human serum	[47]
15	*S. typhimurium*	Multifunctional Au shell-coated graphene oxide nanosheets as a label for SERS signal enhancement	20 cells/mL	20	Milk, vegetable juice, and fruit juice	[53]
16	*S. typhimurium*	Gold-ruthenium nanocomposites	9.8 × 10^4^ CFU/mL	10	Drinking water, orange juice, and milk	[20]
17	*S. typhimurium*	Platinum-coated gold nanorods, magnetic Fe_3_O_4_ nanoparticles	50 CFU/mL (color mode), 75 CFU/mL (magnetic mode)	15	Milk	[52]
18	*S. typhimurium*	CoFe_2_O_4_ having catalase-like activity and magnetic properties	10^2^ CFU/mL	15	Milk and beef	[28]
19	*S. enteritidis*	Glucan-functionalized two-dimensional transition metal dichalcogenides tungsten disulfide as SERS-probe	10^3^ CFU/mL	10	Milk and drinking water	[48]
20	*S. typhimurium*	Au-Fe_3_O_4_ multifunctional nanoparticles	5 × 10^5^ CFU/mL (visual); 5 × 10^4^ CFU/mL (photothermal)	15	Milk	[50]

## Data Availability

The original contributions presented in the study are included in the article and Supplementary Materials; further inquiries can be directed to the corresponding author.

## Supplementary Materials

The following supporting information can be downloaded at: https://www.mdpi.com/article/10.3390/nano13233074/s1, Figure S1: Results of the ELISA optimization: calibration curves of *S. typhimurium* obtained at concentrations of the immobilized Mab of 2 (1), 1 (2), 0.5 (3), and 0.25 (4) µg/mL (a), calibration curves of *S. typhimurium* obtained at concentrations of the biotinylated Mab of 4 (1), 2 (2), 1 (3), and 0.5 (4) µg/mL (b); Figure S2: (a) Images of the test strips after the ICA of pathogens at cell concentration of 10^7^ CFU/mL and (b) images of the test strips after the ICA of *S. tiphymurium* (10^7^ CFU/mL) using Au@Pt conjugated with nonspecific MAb (a) and non-conjugated Au@Pt (b). 1—*S. typhimurium*, 2—*Salmonella paratyphi* A56, 3—*Salmonella Virchov* 06, 4—*Salmonella enteritidis* 3–2, 5—*Salmonella anatum* 1120, 6—*Escherichia coli* 0157:H7 ATCC51658, 7—*Listeria monocytogenes* ATCC51658, 8—*Yersinia enterocolitica* H-26-04, 9—*Yersinia pseudotuberculosis* 4320, 10—*Pseudomonas aeruginosa* ATCC27853, 11—*Francisella tularensis holarctica* 15; Table S1: Liquid flow program of AF4; Table S2: Parameters varied during optimization of the ELISA and the ICAs of *Salmonella typhimurium*; Table S3: Selection of sample preparation technique for milk.
