# Supplementary material for: Sensitive Immunochromatographic Determination of Salmonella typhimurium in Food Products Using Au@Pt Nanozyme
Source: Nanomaterials (Basel). 2023 Dec 4;13(23):3074. doi: 10.3390/nano13233074 (PMC10708492; doi:10.3390/nano13233074)
Supplement: Supplementary file 1 [file nanomaterials-13-03074-s001.zip › nanomaterials-2725326-supplementary.pdf]

nanomaterials

Article

Sensitive immunochromatographic determination of *Salmonella typhimurium* in food products using Au@Pt nanozyme

Olga D. Hendrickson, Nadezhda A. Byzova, Irina V. Safenkova, Vasily G. Panferov, Boris B. Dzantiev and Anatoly V. Zherdev*

A.N. Bach Institute of Biochemistry, Research Center of Biotechnology of the Russian Academy of Sciences, Leninsky Prospect 33, 119071 Moscow, Russia; odhendrick@gmail.com (O.D.H.); nbyzova@inbi.ras.ru (N.A.B.); saf-iri@yandex.ru (I.V.S.); panferov-vg@mail.ru (V.G.P.); dzantiev@inbi.ras.ru (B.B.D.)
* Correspondence: zherdev@inbi.ras.ru; Tel.: +7-495-954-28-04

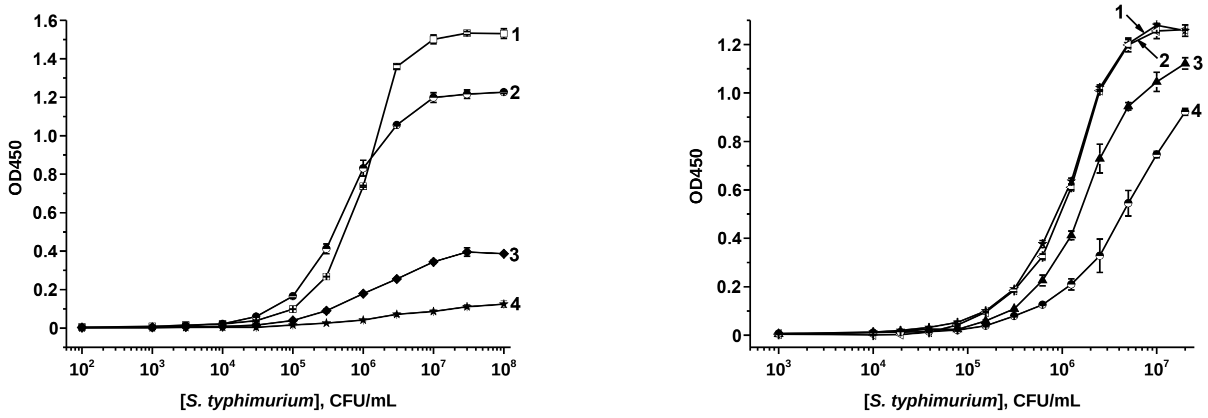

(**a**) (**b**)

Figure S1. Results of the ELISA optimization: calibration curves of *S. typhimurium* obtained at concentrations of the immobilized Mab of 2 (1), 1 (2), 0.5 (3), and 0.25 (4) µg/mL (**a**), calibration curves of *S. typhimurium* obtained at concentrations of the biotinylated Mab of 4 (1), 2 (2), 1 (3), and 0.5 (4) µg/mL (**b**).

Citation: To be added by editorial staff during production.

Academic Editor: Firstname Lastname

Received: date
Revised: date
Accepted: date
Published: date

Copyright: © 2023 by the authors. Submitted for possible open access publication under the terms and conditions of the Creative Commons Attribution (CC BY) license (https://creativecommons.org/licenses/by/4.0/).

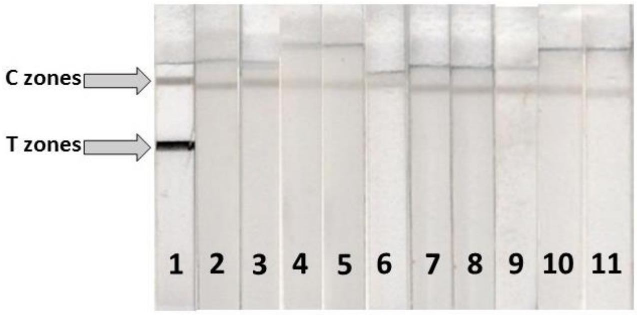

(**a**)

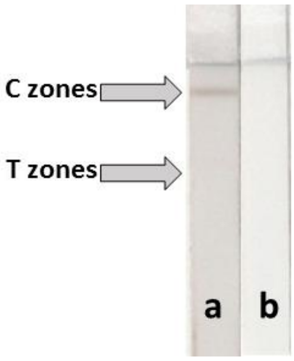

(**b**)

Figure S2. Images of the test strips after the ICA of pathogens at cell concentration of 10^7 CFU/mL (**a**) and images of the test strips after the ICA of *S. tiphymurium* (10^7 CFU/mL) using Au@Pt conjugated with nonspecific MAb (a) and non-conjugated Au@Pt (b) (**b**). 1 – *S. typhimurium, 2 – Salmonella paratyphi* A56, 3 – *Salmonella Virchov* 06, 4 – *Salmonella enteritidis* 3-2, 5 – *Salmonella anatum* 1120, 6 – *Escherichia coli* 0157:H7 ATCC51658, 7 – *Listeria monocytogenes* ATCC51658, 8 – *Yersinia enterocolitica* H-26-04, 9 – *Yersinia pseudotuberculosis* 4320, 10 – *Pseudomonas aeruginosa* ATCC27853, 11 – *Francisella tularensis holarctica* 15.

Table S1. Liquid flow program for AF4.

Stage	Mode	Duration, min	Cross flow, mL/min
1	Elution	2	1.6
2	Focus	2	--------------
3	Focus + Injection	2	--------------
4	Focus	6	--------------
5	Elution	5	1.6
6	Gradient elution	5	1.6–0.4
7	Elution	10	0.4
8	Gradient elution	3	0.4–0.1
9	Elution	3	0.0
10	Elution	3	1.6

Table S2. Parameters varied during optimization of the ELISA and the ICAs of *Salmonella typhimurium*.

Parameter	Range of variation	Selected value
**ELISA**		
Concentration of the immobilized Mab, mg/mL	0.25–2	1
Concentration of the biotinylated Mab, mg/mL	0.5–4	1
**AuNPs-based ICA**		
Concentration of the immobilized Mab, mg/mL	0.25–2	1
Concentration of the immobilized GAMI, mg/mL	0.2–0.6	0.5
OD_{520} of the Mab–AuNPs conjugate	1–8	6
Assay duration, min	5–15	10
**Au@Pt-based ICAs**		
Concentration of the immobilized Mab, mg/mL	0.75–2.5	2
Concentration of the immobilized GAMI, mg/mL	0.2–0.6	0.5
Dilution of the Mab–Au@Pt conjugate, times	10–20	13
Assay duration, min	5–15	10
Volume of the added DAB, μL	1–25	1
Duration of the catalytic stage, min	1–5	2

Table S3. Selection of sample preparation technique for milk.

Parameter	Buffer	Milk of 4% fat content			
		1:5	1:10	1:20	1:25
Signal intensity at 10^8 CFU/mL	36600	n/d**	n/d	32100	34800
IC_{50}*, CFU/mL	4.0×10^6	n/d	n/d	2.0×10^7	3.8×10^6
	**Buffer**	**Milk of 3.2% fat content**			
		1:5	1:10	1:20	1:25
Signal intensity at 10^8 CFU/mL, RU	36800	34200	34600	36000	n/d**
IC_{50}, CFU/mL	4.3×10^6	1.1×10^6	1.0×10^7	4.2×10^6	n/d
	**Buffer**	**Milk of 1.5% fat content**			
		1:5	1:10	1:20	1:25
Signal intensity at 10^8 CFU/mL	36200	30400	35800	n/d	n/d
IC_{50}, CFU/mL	3.7×10^6	5.4×10^6	4.0×10^6	n/d	n/d

*IC_{50} – concentration corresponded to 50% binding with the immobilized MAb

**n/d – not determined
